# Utilizing Apple Pomace in Meat Products: A Systematic Review and Meta-Analysis

**DOI:** 10.3390/foods15091545

**Published:** 2026-04-29

**Authors:** Aigerim Koishybayeva, Yasin Uzakov, Shynar Kenenbay, Malgorzata Korzeniowska

**Affiliations:** 1 Department of Food Technology, Almaty Technological University, 050012 Almaty, Kazakhstan; aigerim.koishybayeva@atu.edu.kz (A.K.); ya.uzakov@atu.edu.kz (Y.U.); 2 Department of Functional Food Products Development, Wroclaw University of Environmental and Life Sciences, 50-375 Wroclaw, Poland; malgorzata.korzeniowska@upwr.edu.pl

**Keywords:** apple pomace, fruit by-products, total dietary fiber, color characteristics, pH, meat products, valorization

## Abstract

Apple pomace, a byproduct of juice production, is a sustainable source of dietary fiber and polyphenols with potential in food systems. This study aimed to systematically review 69 articles and perform a meta-analysis on 17 experimental studies to quantify the impact of AP on meat products. Using a random-effects model (Inverse Variance method; I^2^ = 99–100%), mean differences (MD) and 95% confidence intervals (CI) were calculated. Results revealed that AP significantly increased total dietary fiber (MD = 1.84; *p* < 0.00001) and reduced pH (MD = −0.18; *p* < 0.00001). Regarding color, AP significantly decreased redness (a*) (MD = −1.47; *p* < 0.005) but had no significant impact on lightness (L*) (MD = 0.34; *p* = 0.70) or yellowness (b*) (MD = −1.32; *p* = 0.08). Sensitivity analysis confirmed the robustness of these trends across diverse meat matrices. Despite high statistical heterogeneity, the consistent direction of effect provides high certainty of evidence. Findings suggest that inclusion levels exceeding 10% may increase variability and adversely affect redness and acidity. In conclusion, AP is a promising functional ingredient for sustainable, fiber-enriched meat products. However, a successful application requires optimizing inclusion levels to balance technological performance with consumer acceptance.

## 1. Introduction

The zero-waste concept, which promotes the valorization of fruit and vegetable processing by-products, has become increasingly important in the agri-food sector [1]. Approximately one-third of harvested apples are converted into various products, such as juice, cider, alcoholic beverages, sauces, canned apples, dried apples, and frozen slices [2]. During this process, 25% of the apple’s weight, consisting of peel, core, seeds, stem, and residual pulp, is discarded as apple pomace [2]. It is estimated that the apple processing industry produces up to 10 million tons of apple pomace annually, posing significant environmental and economic challenges in its disposal [3,4]. Apple pomace is, however, a valuable source of dietary fiber, pectin, polyphenols, and other bioactive compounds with demonstrated antioxidant and antimicrobial properties. Due to this composition, it has attracted increasing attention as a dietary supplement, functional food ingredient, or food additive [4]. In particular, recent studies have explored its incorporation into meat products to enhance nutritional value, improve oxidative stability, and extend shelf life. Experimental findings indicate that apple pomace and its derivatives can increase dietary fiber content [5,6,7,8,9], inhibit lipid oxidation, and reduce microbial growth in various meat systems, including beef, pork, chicken, and turkey products [10,11,12,13,14,15]. Previous reviews and meta-analyses on apple pomace have primarily addressed its utilization across a wide range of food systems, including bakery, dairy, and beverage products, with only limited consideration of meat applications [5,16,17]. However, meat systems differ fundamentally from other food matrices due to their complex protein–fat structure, water-binding properties, and susceptibility to lipid oxidation. To address these limitations, the present study conducts a systematic review and meta-analysis of research concerning the incorporation of apple pomace into meat products. By quantitatively integrating experimental data, this study enables a robust statistical evaluation of overall effects and the inherent variability across diverse meat matrices. The primary objective is to determine the impact of varying AP inclusion levels on critical physicochemical and nutritional parameters—specifically pH, total dietary fiber (TDF), and color properties (L*, a*, b*). Furthermore, this study aims to identify the drivers of statistical heterogeneity, providing a scientific foundation for the sustainable fortification of meat products with apple-processing byproducts.

## 2. Materials and Methods

### 2.1. Literature Search and Selection Process

This systematic review and meta-analysis were conducted in accordance with the PRISMA 2020 (Preferred Reporting Items for Systematic Reviews and Meta-Analyses). A comprehensive literature search was performed from 1 January to 15 February 2026 across the Web of Science, Scopus, and Google Scholar databases to identify research published in English between 2000 and 2026. The keywords used in Web of Science and Scopus were “apple AND pomace AND (fortif* OR enrich* OR additive*)”; the search results were screened manually based on article titles. The Google Scholar database was searched using a title-specific query with the search term “allintitle: apple AND [pomace OR peel OR by-product OR fortification OR fiber]”. The reference lists of articles were screened to identify additional relevant articles. After removing duplicate records, study selection was conducted independently by the lead reviewer using a standardized workflow.

### 2.2. Eligibility Criteria

The following eligibility criteria were applied for study selection: (1) original experimental research involving meat products; (2) incorporation of AP or its derivatives; and (3) provision of quantitative data (means and variance) for pH, total dietary fiber (TDF), and color parameters (L*, a*, b*). Studies were excluded if they lacked a 0% fortification control group, failed to report variance data (SD or SE), or utilized non-standardized analytical methodologies.

### 2.3. Data Extraction and Risk of Bias Assessment

Data extraction was conducted by the lead reviewer using a structured data collection form to ensure consistency. Extracted parameters included author information, meat species, inclusion levels of apple pomace, and quantitative outcomes.

The risk of bias for the 17 included studies was evaluated based on four quality indicators: (1) the inclusion of a 0% AP control group; (2) the execution of experiments in at least triplicate; (3) the use of standardized analytical procedures for pH, color, and TDF; (4) the provision of clear variance data (standard deviation, SD; or standard error, SE). Studies meeting all criteria were categorized as having a low risk of bias. A detailed PRISMA checklist is provided in Supplementary Table S1.

### 2.4. Statistical Analysis and Synthesis Methods

Meta-analysis was conducted using RevMan Software (Cochrane Collaboration, 5.4. London, UK). Given the high anticipated heterogeneity across meat matrices and inclusion levels, a random-effects model was utilized via the Inverse Variance (IV) method to calculate pooled mean differences (MD) and 95% confidence intervals (CI). Data preparation involved converting SE to SD where necessary, using the formula: SD = SE$\sqrt{n}$. For studies reporting multiple inclusion levels, each level was treated as an independent comparison against the control.

Statistical heterogeneity was quantified using the I^2^ statistic and Chi^2^ test, with I^2^ > 75% representing high heterogeneity. To evaluate the robustness of the findings, a “leave-one-out” sensitivity analysis was performed. Potential reporting bias (publication bias) was assessed through visual inspection of funnel plots (Supplementary Figures S1–S4) for outcomes with *n* ≥ 10 comparisons.

#### Certainty of Evidence

The certainty of the evidence for each outcome (pH, TDF, and color) was qualitatively assessed based on four domains: (1) risk of bias in the primary studies; (2) consistency of results across different meat matrices (I2); (3) precision of the pooled effect estimates (95% CI); and (4) directness of the evidence in relation to the research objectives. The overall confidence in the results was further supported by the statistical significance of the pooled effect sizes (*p* < 0.05).

## 3. Results

### 3.1. Study Selection and Flow Diagram

**Figure 1:**
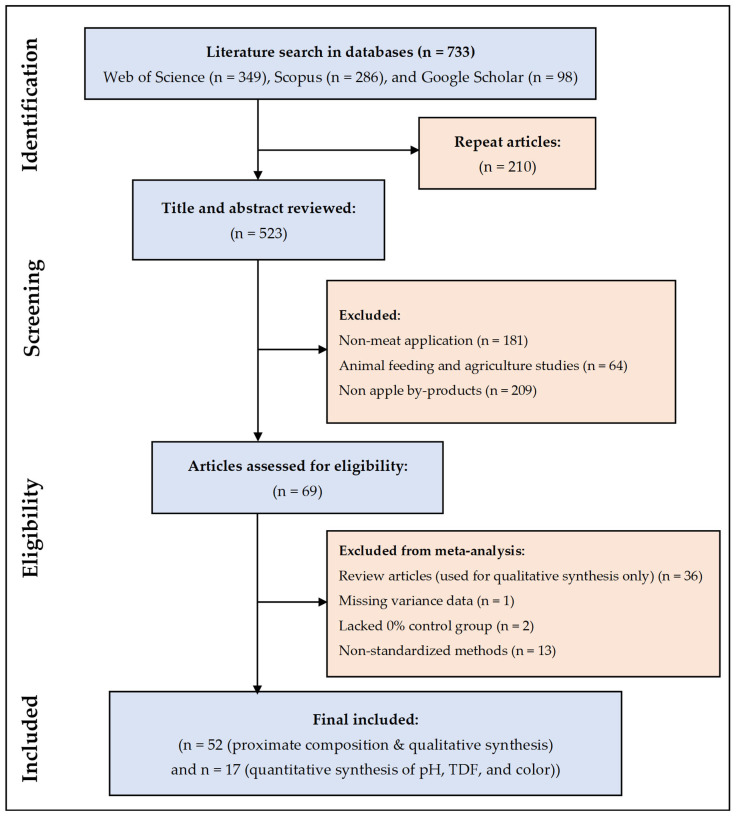
PRISMA flow diagram of the literature search.

The systematic search across databases initially identified 733 records. After removing duplicates and screening titles and abstracts, 69 full-text articles were assessed for eligibility. Ultimately, 69 articles were included in the systematic review, of which 17 provided sufficient quantitative data for meta-analysis (Figure 1). Studies excluded at the full-text stage were primarily omitted due to a lack of standard deviation (SD) reporting and the absence of a proper 0% fortification control group.

### 3.2. The Proximate Composition of Apple Pomace

The composition of apple pomace varies significantly and is influenced by factors such as the manufacturing process, apple type, harvest year, specific processing techniques, and drying method. It is recognized as a potential ingredient with antioxidant, anti-inflammatory, antibacterial, and antiviral properties [16,18].

The proximate composition of apple by-products in comparison with whole apple is presented in Table 1. Fresh apple pomace, with a substantial moisture content ranging from 66.4% to 78.2%, is highly susceptible to spoilage [19]. Additionally, its high sugar content, comprising glucose, fructose, and sucrose, accounts for 48.0–62.0% of its composition [20]. Pomace, characterized by its high moisture content, requires preservation through drying or, less commonly, freezing [21]. When used in its dried form, pomace increases the total dietary fiber and polyphenol levels. Studies have indicated that drying fruit pomace increases the concentration of extractable phenolic compounds, resulting in a 58% increase in total phenolic content compared with the moist form [20]. Lowering the moisture content to approximately 10% or less by drying effectively inhibits most microorganisms from proliferating and prevents the degradation of phytochemicals during storage. This process significantly extends the shelf life and allows for use beyond the apple harvest period [20,22]. Freeze-drying is considered the optimal method for maintaining the original appearance and nutrients of fresh materials [23]. However, the cost of freeze-drying can be four to eight times higher than that of traditional hot-air drying methods [20]. The drying conditions had a notable impact on the color characteristics of the dried samples, as evidenced by a clear reduction in L* and a* values, while the E* value increased [24]. A greater amount of apple pomace typically resulted in a darker appearance of the meat analogs, as indicated by lower L* values. This darkening can be attributed to melanoidins, brownish-red pigments formed through the Maillard reaction, which reduce lightness [25]. Conversely, in the study by Ma et al., the L* value of the freeze-dried sample was slightly higher than that of the fresh sample, whereas the a* and b* values were lower. This could be due to the low temperature and vacuum conditions during freeze-drying, along with the presence of sponge-like tissue voids, which resulted in a lighter appearance [26].

Total dietary fibers are present in notable quantities (45–51%) in apple pomace [32,33] in both soluble and insoluble forms [33,34,35]. Insoluble fibers include cellulose (β-1,4-glycosidic-linked glucoses), hemicelluloses (such as xyloglucan, galactomannan, and glucuronarabinoxylan), and lignin (composed of polymerized coniferyl, sinapyl, and p-coumaryl alcohols) [36]. Research examining the fiber content in whole apples, their pulp, and their peel has revealed that the peel contains the highest total fiber [27,37]. Various drying techniques can alter the dietary fiber content in apple peels, which ranges from 34.22 g/100 g to 38.71 g/100 g [26]. Additionally, smaller particle-size fractions had significantly lower fiber content, ranging from 27.9 to 32.0 g/100 g d.w., compared to the coarser fractions in apple pomace flour, where the fiber content ranged from 34.9 to 37.7 g/100 g d.w. [38].

Together with dietary fibers, phenolic and polyphenolic compounds are the most important constituents of apple pomace. It contains a wide array of natural antioxidants, including quercetin glycosides, phloridzin, and other phenolic substances known for their potent antioxidant properties [39]. Cider apples have a higher phenolic content than dessert apples [28]. Typically, smaller and biennial cider apples, which are often bitter, possess more total phenolics than dessert apples, which are primarily consumed fresh or used for juice production [40]. Among the 40 apple varieties, the total phenolic content in fresh fruits ranged from 66.2 to 211.9 mg/100 g, highlighting the diversity of polyphenolic content among apple varieties. Catechins and proanthocyanidins are the predominant fractions in apple pomace, followed by hydroxycinnamates, flavonols, dihydrochalcones, and anthocyanins [40]. The seeds have a significantly higher total phenolic content (651.80 mg/100 g d.w.) than the pulp (25.60 mg/100 g d.w.), with the peel containing 146.29 mg/100 g d.w. [28,41,42,43]. Smaller particle fractions of apple pomace contained the most phenolic compounds, whereas the largest particles had the least (93.0 mg GAE/g d.w.) [38]. This increase in phenolic compounds in finer particles is attributed to the larger surface area and cell wall disruption, which enhances phenolic extraction [44]. The red coloration of apple skins is largely due to anthocyanidins [45], and red-skinned varieties such as “Red Delicious,” “Redfree,” and “Discovery” have higher anthocyanin content than yellow or green-skinned apples [28,46]. For instance, the green-skinned “Granny Smith” apple peel contains only 0.12 mg/kg of cyanidin 3-O-galactoside, whereas the red-skinned “Story” apple contains 315 mg/kg [47].

Approximately 82% of the total polyphenols are retained in apple pomace following fruit processing [16]. Apple pomace is rich in polyphenols, comprising 31–51%, with a notable presence of cinnamate esters, dihydrochalcones, and flavonols [48]. It also contains a wide array of natural antioxidants, including quercetin glycosides, phloridzin, and other phenolic compounds known for their potent antioxidant properties [39]. Dry apple pomace exhibits a broad range of polyphenol content, measured between 262 and 856 mg of total phenols per 100 g [33,49]. For example, the peels of “Red Delicious” apples contain 1187.0 mg/100 g of total polyphenols, which is four times higher than the 304.0 mg/100 g found in “Granny Smith” apple peels [50]. Flavan-3-ols and flavonols, along with most flavonols and anthocyanidins, are predominantly found in apple peels [51,52], while newly developed red-flesh apple varieties have a high concentration of flavonols and anthocyanidins in their flesh [46,47]. The milling process enhances the antioxidant content, balancing oxygen consumption and thereby increasing the antioxidant capacity, which remains dynamically stable over time. This could be due to the prevention of oxidation by minimizing the apple pomace suspension’s exposure to oxygen during milling [53].

Carbohydrates, which make up 71% [16,54], are primarily composed of insoluble sugars such as cellulose (127.9 g/kg d.w.), hemicellulose (ranging from 7.2 to 43.6 g/kg d.w.) [34,55], and lignin (15.3 to 23.5 g/kg d.w.). Additionally, simple sugars such as glucose (22.7%), fructose (23.6%), and galactose (6–15%) [56,57], as well as pectin [58], are present. The polysaccharides in apple pomace include α-(1-4)-linked d-galacturonic acid units (49–64%), arabinose (14–23%), galactose (6–15%), and smaller amounts of rhamnose, xylose, and glucose [59]. Washing apple pomace decreased total sugar from 55.2% to 32.6%, fructose from 31.3% to 17.9%, glucose from 12.6% to 8.6%, and sucrose from 11.3% to 6.1% [60]. Grinding apple pomace into powder results in a higher carbohydrate content, likely due to the increased specific surface area of the finer particles, which facilitates the release of soluble sugars during extraction [38]. The levels of total sugar and titratable acidity can influence the flavor and taste of products incorporating apple by-products as dietary supplements [26]. Depending on the drying method, the total sugar content of apple pomace can either increase or decrease; for instance, the highest and lowest total sugar contents were observed in samples dried by air at 75 °C (506.68 mg/g) and by heat pump at 65 °C (431.68 mg/g) [26].

Apple pomace also contains minerals such as sodium, phosphorus, potassium, manganese, calcium, magnesium, zinc, copper, and iron [2,27,38,59,61]. Moreover, apple peels contain more minerals than whole apples [2], especially higher levels of zinc, iron, and copper were present in the peel than in the apple flesh [37].

According to Skinner et al., apples are the second most vitamin-rich fruit after cranberries. Apple pomace is a potent source of antioxidants and is rich in vitamins A, C [34], and E [2,62]. Apple seeds contain more vitamins than other apple parts [63]. The vitamin C content in apple peels (2.7 to 56.0 mg/100 g of fresh weight) is double that found in the inner parts (0.1–13.9 mg/100 g of fresh weight) [64,65]. Apples also contain trace amounts of vitamins B12 and D [2]. As the particle size of apple pomace decreased, the vitamin E content dropped from 2.0 to 1.5 mg/100 g, likely due to degradation during air-drying, which is intensified by increased exposure to air and heat [38]. Conversely, reducing particle size, especially to less than 1 mm, generally increases water-soluble vitamin levels. This is likely due to improved extraction efficiency from increased surface area [66].

### 3.3. Systematic Review of Apple Pomace as a Fortifying Agent

Apple pomace has been used to fortify meat products. The studies included burgers, meatballs, nuggets, sausages, patties, baked pork, salami, and chevon rolls. A summary of the above-mentioned studies is presented in Table 2.

Studies on the fortification of meat products mainly aimed to investigate the effects of the inclusion of apple by-products on the nutritional profiles, shelf life, and techno-functional properties. Studies that included meat products as fortified products used wet apple pomace, ultrasound-treated apple pomace, dried apple pomace (powder, flour), apple pulp, apple peel, and apple flesh for fortification. Inclusion level is a key factor influencing the variability of results. Lower levels of apple pomace tend to provide functional benefits without adversely affecting meat product quality, whereas higher levels may cause undesirable changes in texture and sensory properties.

Several studies included nuggets as fortified products [73,78,81]. For example, Verma et al. analyzed chicken nuggets where lean meat was replaced with 8, 10, and 12 g/100 g of apple pulp [81]. Across several studies, a consistent finding was that higher concentrations of apple pomace, such as those above 8% in chicken nuggets, frequently led to decreased emulsion stability and cooking yield, despite increasing dietary fiber content. The final fortified products were soft. Sensory evaluation revealed a significant reduction in the texture and overall acceptability scores of the treatment products. Yadav et al., 2016b [73] prepared chicken nuggets by replacing lean meat with 3, 6, and 9% apple pomace. Emulsion stability, cooking yield, and fiber content increased significantly in the treated nuggets; however, the sensory acceptability was lower than that of the control. The authors concluded that dietary fiber-enriched chicken nuggets with very good acceptability can be prepared by incorporating 6% dried apple pomace. The effect of fortification was similar to that reported by Huda et al. [78], with increases in dietary fiber and changes in texture observed. The addition of 5% apple pomace was found to be more acceptable to consumers.

Fortification of sausages has also been an interesting topic in studies [67,68,70,72,74,75]. Thangavelu et al. [67] used ultrasound-treated apple pomace in sausages as phosphate replacers. The results showed that the addition of ultrasound-treated apple pomace to the formulation produced a significant interaction effect, increasing water-holding capacity and emulsion stability and decreasing cooking loss. However, the TBARS values increased on day 9. No significant changes were observed in the color, texture, or proximate content values. A different approach was taken by Choi et al. [68], who added apple pomace fiber at concentrations of 1 and 2% to chicken sausages. An increase in fiber content stability and a decrease in redness were observed in the fortified products. The addition of apple pomace fiber successfully reduced the fat content in emulsion sausages while improving their quality characteristics compared to the control. The replacement of lean meat with 3–9% apple pomace was studied by Yadav et al. (2016a) [72], Jandyal et al. [74], and Koishybayeva & Korzeniowska [75] in chicken, pork, and turkey sausages, respectively. In all studies, fiber content, emulsion stability, and cooking yield increased. Higher concentrations of apple pomace led to a mild, sweet flavor in treated pork sausages, and a 6% rate was recommended for the high-fiber sausage without affecting sensory attributes. Koishybayeva and Korzeniowska [75] observed an increase in the total phenolic content and antioxidant activity of fortified products. The addition of apple pomace negatively affected color; however, sensory analysis revealed that 3% apple pomace yielded marks very similar to the control sample. In another study by Yadav et al. [70], a high dose of apple pomace adversely affected the textural properties and color change in treated chicken sausages compared with the control. In conclusion, chicken sausage with very good sensory acceptability can be prepared using 4% apple pomace.

Studies on the use of apple pomace to enrich buffalo meat patties have also been performed. Younis and Ahmad [71] used apple pomace to replace meat at 2%, 4%, 6%, and 8%. The results revealed increases in water-holding capacity, cooking yield, emulsion stability, and crude fiber content. The addition of apple pomace influenced the texture and color of the treated patties. However, only an addition of up to 6% apple pomace was acceptable to the panelists.

Goshtaba was also studied when fortified with apple pomace powder at concentrations of 1, 3, and 5% as a fat replacer [69]. The addition of apple pomace increased cooking yield and color properties, and reduced diameter and thickness compared with the control sample. The TBARS values were clearly lowered at high concentrations of apple pomace. The sensory analysis of goshtaba with 1 and 3% apple pomace was similar to that of the control sample.

Pollini et al. [76] enriched beef burgers with 4 and 8% apple pomace. The addition of apple pomace increased the burgers’ fiber content, thus it contains 40.19% of dietary fiber. The color and sensory analysis of the fortified burgers was even better than that of the control. Ultrasound-assisted extraction improved the phenol content of fortified burgers. Sensory analysis revealed higher palatability for consumers who did not like meat.

Grispoldi et al. [80] used two concentrations of apple pomace to enrich Italian salami. The addition of apple pomace increased fiber and phenol content and lowered fat and calorie content, representing the most interesting characteristics of fortified salami. There was a slight difference in overall acceptability, and the two formulations were an interesting approach to adding healthy compounds to salami.

Keska et al. [22] studied the fortification of meat products with 0.5 and 1% apple peel and flesh. The addition of 0.5% apple peel or 1% apple flesh was most effective at inhibiting lipid oxidative changes in baked meat products, reducing the TBARS index from 47.5% to 39.04%. Even at low concentrations, apple pomace affected the fatty acid profile of meat products relative to the control sample.

Kurmanbekova et al. [79] enriched chicken meat by adding 5, 10, 15, 20, 25, and 30% apple by-products. The addition of apple by-products reduced cooking loss and malondialdehyde formation in treated chicken meat. The inclusion of 5%, 10%, and 15% apple by-products did not impair the quality of chicken meat, despite color changes. Sensory analysis revealed that 15% treated chicken meat was favorably accepted and had a higher content of essential free amino acids than the control.

Gracey et al. [8] used apple pomace at two levels (10 and 20%) to enrich beef meatballs. The fortification was successful in terms of the fiber content. However, increasing the amount of apple pomace decreased the meatball cooking yield. No significant changes were observed in the sensory attributes or texture analyses.

The addition of 6% apple pomace in the study by Parkash et al. [77] reduced lipid oxidation in chevon rolls. A decrease in texture was observed for the treated rolls. In addition, the decrease in the meaty flavor of the treated rolls resulted in low acceptability among panelists, as evidenced by lower flavor scores.

### 3.4. Meta-Analysis and Quantitative Synthesis

The quantitative synthesis was performed to determine the pooled effect of AP on pH, TDF, and color parameters. High heterogeneity was expected and accounted for using a random-effects model.

**Figure 2:**
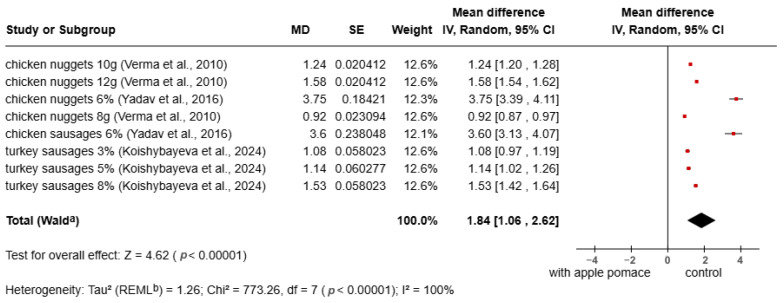
Meta-analysis of TDF in fortified products [72,73,75,81].

The meta-analysis of total dietary fiber (TDF) in meat products enriched with apple pomace is presented in Figure 2. The results showed a significant increase in TDF content in meat products with apple pomace compared to the control group (MD = 1.84; 95% CI: 1.06–2.62; *p* < 0.00001). All included studies demonstrated a positive effect, with values to the right of the null line (MD = 0), indicating higher TDF levels after fortification. The increase in TDF is due to the high TDF content (45–51%) in apple pomace [32,33].

**Figure 3:**
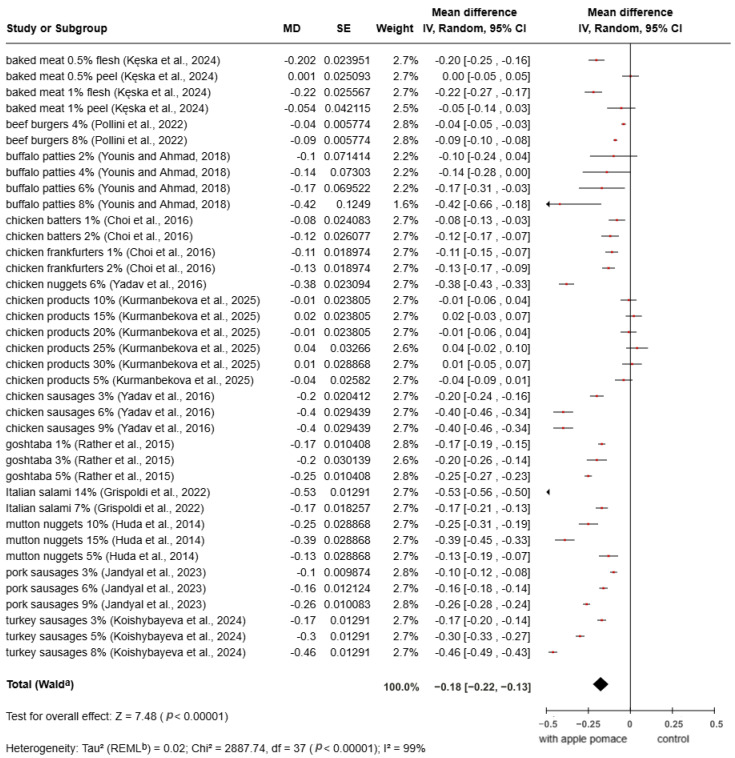
Meta-analysis of pH value in fortified products [22,68,69,71,72,73,74,75,76,78,79,80].

The pH results from different studies that included product fortification with apple pomace are summarized in Figure 3. The results showed a statistically significant decrease in pH in samples containing apple pomace compared to the control group (MD = −0.18; 95% CI: −0.22 to −0.13; *p* < 0.00001). Most studies showed negative MD values, indicating that the addition of apple pomace reduced the pH of meat products, except for 15%, 25%, and 30% chicken products [79]. The reduction in pH observed with the incorporation of apple pomace may contribute to improved microbial stability and shelf life, but excessive decreases could negatively affect texture, color, and sensory properties. Therefore, the level of apple pomace addition should be carefully optimized.

**Figure 4:**
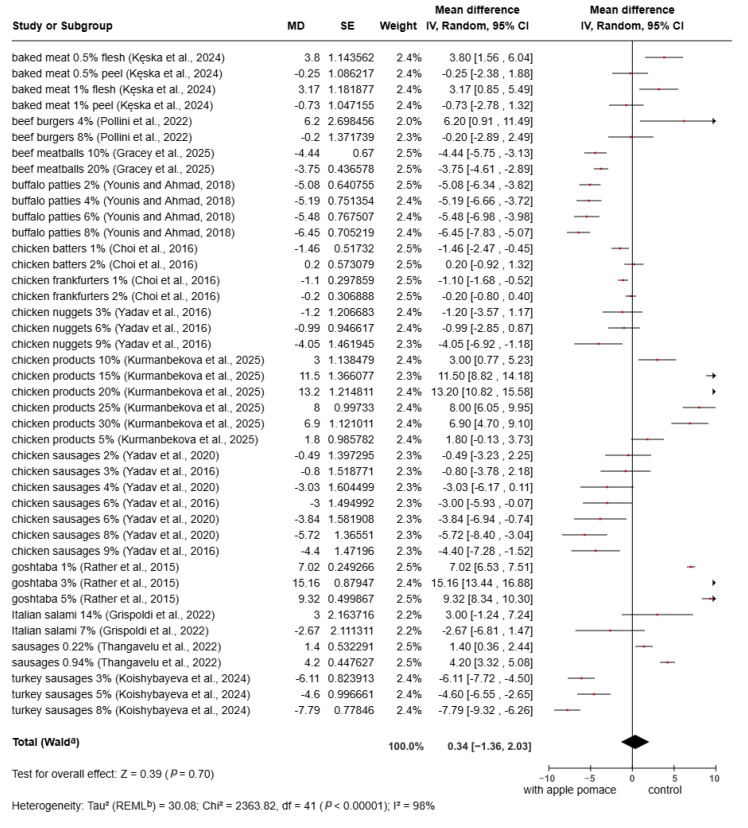
Meta-analysis of L* value in fortified products [8,22,67,68,69,70,71,72,73,75,76,79,80].

The meta-analysis of the L* value is presented in Figure 4. The results indicate no significant effects for L* values in products containing apple pomace compared to the control group (MD = 0.34; 95% CI: −1.36 to 2.03; *p* = 0.70). However, individual studies showed both positive and negative effects, suggesting that the impact of apple pomace on color varies across studies. The color, in terms of L* value, was influenced by the degree of concentration, the type of meat product, the processing method, and the type of apple pomace used [82]. The darker color of meat products is due to the Maillard reaction, where fructose reacts with proteins during cooking, causing browning [25]. An increase in L* values may enhance visual appeal in some products; however, excessive changes in lightness could negatively affect consumer acceptance, depending on the product type and expected color characteristics.

**Figure 5:**
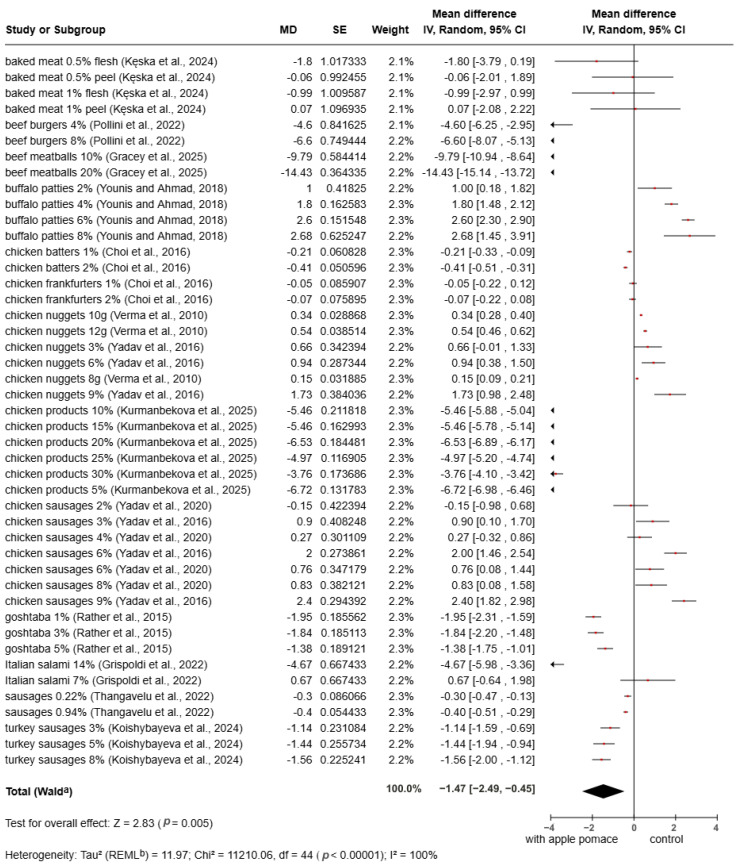
Meta-analysis of a* value in fortified products [8,22,67,68,69,70,71,72,73,75,76,79,80,81].

The results of the meta-analysis of a* values are summarized in Figure 5. The overall pooled result showed a significant decrease in a* values in samples containing apple pomace compared with the control (MD = −1.47, 95% CI: −2.49 to −0.45, *p* = 0.005). The red color in apple pomace originates from carotenoids, natural pigments that impart yellow, orange, and red hues to fruits and vegetables [83]. The negative a* value (more green color) may be due to other reactions of polyphenols and carotenoids [16]. The a* value increased with increasing apple pomace content. This trend aligns with the findings of Yadav et al. [70], who observed a significant increase in a values in chicken sausages containing 2–8% apple pomace, 2–8% buffalo meat patties [71], 3–9% chicken sausage [72], 3–9% chicken nuggets [73], 7% Italian salami [80] and 8–12% chicken nuggets [81].

**Figure 6:**
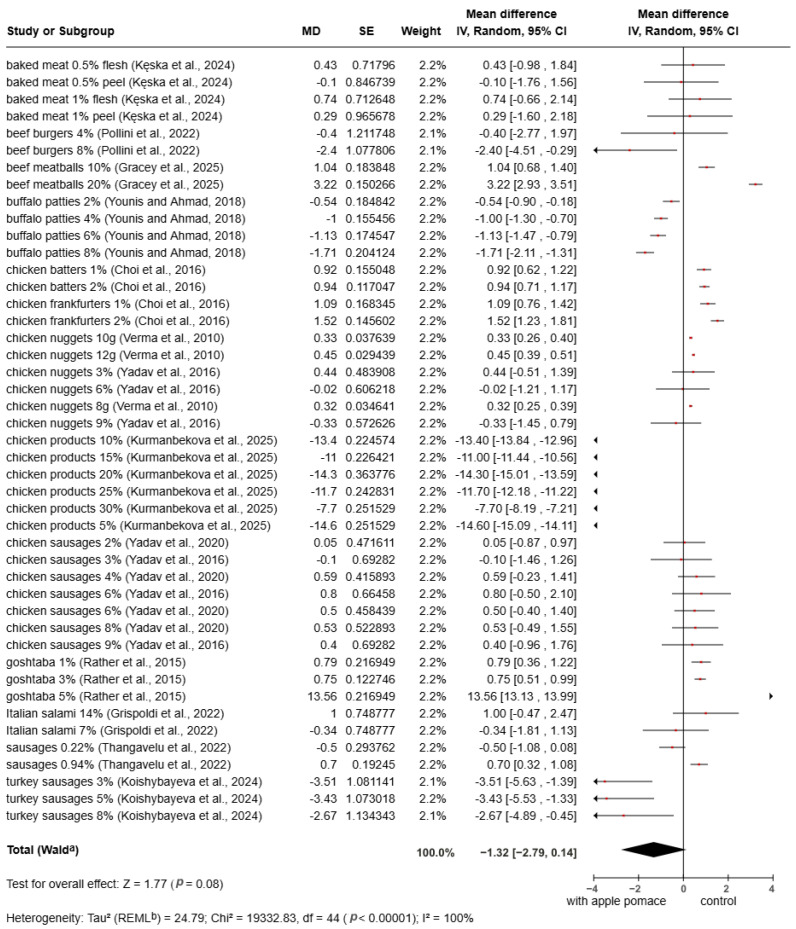
Meta-analysis of b* value in fortified products [8,22,67,68,69,70,71,72,73,75,76,79,80,81].

The meta-analysis of b* color values showed a slight decrease in apple pomace fortified meat products compared to the control. However, this effect was not statistically significant (MD = −1.32, 95% CI: −2.79 to 0.14, *p* = 0.08). While the forest plot (Figure 6) shows a trend toward lower b* values, the 95% confidence interval crosses the line of no effect (zero), indicating that the addition of apple pomace does not consistently alter the yellowness of the final product. The stability of the b* value is an important finding for the technological application of apple pomace. Generally, plant-based additives can increase yellowness due to the presence of carotenoids or flavonoids [69,84].

## 4. General Discussion and Synthesis of Evidence

The results of this synthesis confirm that apple pomace (AP) is a potent functional ingredient for fiber enrichment in meat products, consistently increasing total dietary fiber (TDF) while causing a modest reduction in pH and maintaining relative stability in yellowness (b*), results that are directly supported by its high fiber content (45–51%) and the presence of organic acids. While the meta-analysis reveals significant nutritional and technological benefits, the body of evidence is limited by high statistical heterogeneity (I^2^ > 90%), which likely stems from the diversity of meat matrices (e.g., poultry vs. beef) and unstandardized AP processing methods, such as varying drying temperatures. Additionally, the review process itself has limitations, notably the restriction to English-language publications and the reliance on an independent lead reviewer for screening and data extraction, which may introduce minor selection bias. For the meat industry, these findings imply that AP is a viable, sustainable additive for “high-fiber” labeling, particularly at inclusion levels below 10% to prevent excessive browning or loss of redness; however, future research must prioritize standardized drying protocols and the quantitative synthesis of sensory data to ensure full consumer acceptance and product consistency.

## 5. Conclusions

This systematic review and meta-analysis provide a quantitative assessment of apple pomace (AP) as a functional ingredient in meat products. By synthesizing data from 69 studies for characterization and 17 original studies for meta-analysis, we demonstrate that AP effectively enhances the nutritional profile of meat products. The meta-analysis shows a significant increase in total dietary fiber (MD = 1.84) and a reduction in pH (MD = −0.18), likely driven by the inherent organic acids and fiber content of the pomace. Importantly, our findings address a major industrial concern regarding color stability. While AP incorporation leads to a significant reduction in redness (a*), it maintains the lightness (L*) and yellowness (b*) of the meat products, suggesting that it can be utilized without severely darkening the final product. The high statistical heterogeneity (I^2^ = 99–100%) observed across studies reflects the natural biological variability of different meat matrices and the lack of standardization in pomace processing, rather than uncertainty in the evidence. Our results suggest that inclusion levels up to 10% are generally well tolerated, whereas higher concentrations may require specific formulation adjustments to prevent adverse impacts on color and acidity. Future research should focus on standardizing the chemical profile of AP and exploring the synergistic effects of AP with other natural antioxidants to preserve redness. Ultimately, upcycling apple pomace into meat systems represents a viable strategy for developing sustainable, “clean-label,” and fiber-enriched functional foods.

## Figures and Tables

**Table 1:** The proximate composition of apple by-products in comparison with whole apple [2,6,7,16,27,28,29,30,31].

Compounds	Whole Apple	Apple Pomace	Apple Peel	Apple Seed
Nutritional composition (g/100 g)				
Fat	0.2–0.3	1.2–3.6	2.7–14.8	19.7
Protein	0.1–0.2	1.2–5.9	2.8–4.5	35.3–40.1
Total Carbohydrate	10.0	44.5–57.4	60.0–81.9	19.5–21.1
Total Fiber	2.0–2.1	27.9–49.5	40.3–43.9	3.66–5.20
Complex carbohydrates, %				
Insoluble fiber	1.54	33.8–60.0	42.1	4.12
Soluble fiber	0.67	13.5–14.6	5.8	n.d.
Pectin	0.71–0.93	3.2–13.3	9.84	n.d.
Simple carbohydrates, %				
Fructose	5.8–6.0	44.7	n.d.	n.d.
Glucose	2.4–2.5	18.1–18.3	n.d.	n.d.
Mineral composition (mg/100 g)				
Sodium (mg/100 g)	<1.0–1.0	39.3	<0.01–0.4	n.d.
Potassium (mg/100 g)	59.0–95.0	872.8–925.0	800.0–1100.0	650.0
Calcium (mg/100 g)	0.20–0.50	5.56–9.27	100.0	27.0
Phosphorus (mg/100 g)	6.0–9.0	50.0–112.0	100.0	72.0
Magnesium (mg/100 g)	3.0–4.7	20.0–61.6	100.0	51.0
Iron (mg/100 g)	<0.1–0.1	2.4–23.0	1.3–1.4	11.0
Zinc (mg/100 g)	0.02	0.9–1.8	1.0	4.4
Copper(mg/100 g)	0.01–0.02	0.6–0.9	0.04–0.2	0.2
Manganese (mg/100 g)	0.02–0.03	0.4–1.8	0.02–0.5	0.5
		Vitamins (mg/100 g)		
C	18.5	22.4	n.d.	v.l.
E	<1.0	5.5	n.d.	1290.5
Specific phenolics				
Chlorogenic acid	16.16 mg/L	26–2298 mg/kg	3.9–18.1 mg/100 g	15.74–32.90 mg/100 g
(+)-Catechin	3.33	1–127	1.5–8.3	2.43–9.41
(−)-Epicatechin	23.26	4.2–640	1.1–18.5	4.11–7.43
Procyanidin B1	n.d.	n.d.	0.0–4.8	n.d.
Procyanidin B2	n.d.	48.8–590.2	4.4–9.6	2.25–9.72
Rutin	n.d.	n.d.	0.9–24.3	n.d.
Phloridzin	3.54	8–1435.4	0.8–63.8	282.74–864.42
Total polyphenols	52.53	n.d.	36.39–256.19	313.79–989.82
Coumarin	4.25	n.d.	n.d.	n.d.
Caffeic acid	1.99	3–280	n.d.	n.d.
Quercitrin	n.d.	69–373.8	n.d.	n.d.

n.d.—no data, v.l.—very low.

**Table 2:** Effect of the addition of apple pomace to the meat products.

Study	Meat Type	Pomace Form & Processing	Inclusion Level (%)	Application Type	Positive Effects	Negative Effects
Low level ≤ 3%						
Thangavelu et al. [67] (2022)	Breakfast sausage	Ultrasound-treated; freeze-dried powder	0.22–0.94	Addition	↑ WHC,↑ emulsion stability,↓ cooking loss.	↑ TBARS
Kęska et al. [22] (2024)	Pork (baked)	Freeze-dried powder	0.5–1	Addition	↓ lipid oxidation, ↑ fatty acid profile, stabilized aw	↑ L*,↓ a*,↑ b*, ↑ microbial counts
Choi et al. [68] (2016)	Chicken sausages	Enzyme-treated fiber	1–2	Addition	-	↑ L*,↓ a*
		Medium level 3–10%				
Rather et al. [69] (2015)	Mutton goshtaba	Powder (industrial)	1–5	Fat replacement	↑ yield,↑ a*,↑ b*, ↓ lipid oxidation, stabilized shape	↓ L*,↓ hardness, ↓ acceptability
Yadav et al. [70] (2020)	Chicken sausages	Dried powder	2–8	Meat replacement	↑ a*,↑ springiness, ↑ cohesiveness	↓ sensory acceptability, ↑ hardness
Younis & Ahmad [71] (2018)	Buffalo meat patties	Dried powder	2–8	Meat replacement	↑ yield,↑ emulsion stability,↑ fiber,↑ a*, ↑ firmness,↑ chewiness	↑ hardness ↓ L*,↓ b*, ↓ acceptability
Yadav et al. [72] (2016a)	Chicken sausages	Dried powder	3–9	Meat replacement	↑ emulsion stability, ↑ yield,↑ fiber,↑ a*, ↓ lipid oxidation	↓ L*
Yadav et al. [73] (2016b)	Chicken nuggets	Dried powder	3–9	Meat replacement	↑ fiber,↑ a*	↓ sensory acceptability,↓ L*, ↑ hardness
Jandyal et al. [74] (2023)	Pork sausages	Dried pulp powder	3–9	Meat replacement	↑ yield,↑ emulsion stability,↑ gumminess, ↑ chewiness,↑ cohesiveness, ↓ hardness	↓ acceptability
Koishybayeva & Korzeniowska [75] (2024)	Turkey sausages	Freeze-dried powder	3–8	Meat replacement	↓ cooking loss, ↓ microorganism counts, ↑ TPC,↑ fiber,↑ antioxidant activity	↓ L*,↓ a*,↓ b* ↓ acceptability
Pollini et al. [76] (2022)]	Beef burger	Freeze-dried powder	4–8	Meat replacement	↑ fiber,↑ phenol contents, ↑ palatability	↓ acceptability ↑ LAB
Parkash et al. [77] (2016)	Chevon rolls,	Dried powder	6	Meat replacement	↓ lipid oxidation	↓ acceptability
		High level ≥ 10%				
Huda et al. [78] (2014)	Mutton nuggets	Wet pomace	5–15	Meat replacement	↑ emulsion stability, ↑ yield,↑ dietary fiber, ↑ springiness	↓ hardness, ↓ acceptability
Kurmanbekova et al. [79] (2025)	Chicken meat products	Dried powder	5–30	Addition	↓ cooking loss,↓ MDA	↑ L*,↓ a*,↓ b*, ↑ EAAs, ↑ hardness,
Grispoldi et al. [80] (2022)	Pork salami	Dried powder	7–14	Meat replacement	↑ fiber,↑ TPC, ↓ fat,↓ spoilage bacterial populations	↑ hardness,↓ LAB,↓ a*
Verma et al. [81] 2010	Chicken nuggets	Wet pomace	8–12	Meat replacement	↑ fiber,↑ a*,↑ b*	↓ emulsion stability,↓ yield, ↓ textural properties, ↓ acceptability
Gracey et al. [8] (2025)	Beef meatballs	Freeze-dried powder	10–20	Addition	↑ fiber	↓ a*,↓ yield

↑—positive effect, ↓—negative effect.

## Data Availability

The data presented in this study are available upon request from the corresponding authors due to privacy concerns.

## Supplementary Materials

The following supporting information can be downloaded at: https://www.mdpi.com/article/10.3390/foods15091545/s1, Figures S1–S4: Funnel plots for publication bias assessment; Figure S1: Funnel plot for publication bias assessment of pH values in meat products with apple pomace addition; Figure S2: Funnel plot for publication bias assessment of *L** values in meat products with apple pomace addition; Figure S3: Funnel plot for publication bias assessment of *a** values in meat products with apple pomace addition; Figure S4: Funnel plot for publication bias assessment of *b** values in meat products with apple pomace addition; Table S1: PRISMA checklist.

## Abbreviations

The following abbreviations are used in this manuscript:

AP	Apple pomace
TBARS	Thiobarbituric Acid Reactive Substances
TDF	Total Dietary Fiber
TPC	Total Phenolic Content
*L**	Lightness
*a**	Changes in the color from green to red
*b**	Changes in the color from blue to yellow
d.w.	Dry weight
MDA	Malondialdehyde
LAB	lactic acid bacteria
EAAs	free essential amino acids
MD	Mean Difference
CI	Confidence Interval
SD	Standard Deviation
SE	Standard Error
IV	Inverse Variance
REML	Restricted Maximum Likelihood
I^2^	I-squared (Statistic for heterogeneity)
Chi^2^	Chi-squared (Test for statistical significance of heterogeneity)
Z	Z-score (Test for overall effect)
*p*	*p*-value (Statistical significance)
df	Degrees of Freedom
n	Number of samples/comparisons
