# Supplementary material for: Utilizing Apple Pomace in Meat Products: A Systematic Review and Meta-Analysis
Source: Foods. 2026 Apr 29;15(9):1545. doi: 10.3390/foods15091545 (PMC13164446; doi:10.3390/foods15091545)
Supplement: Supplementary file 1 [file foods-15-01545-s001.zip › Figure S1-4. Funnel plots.pdf]

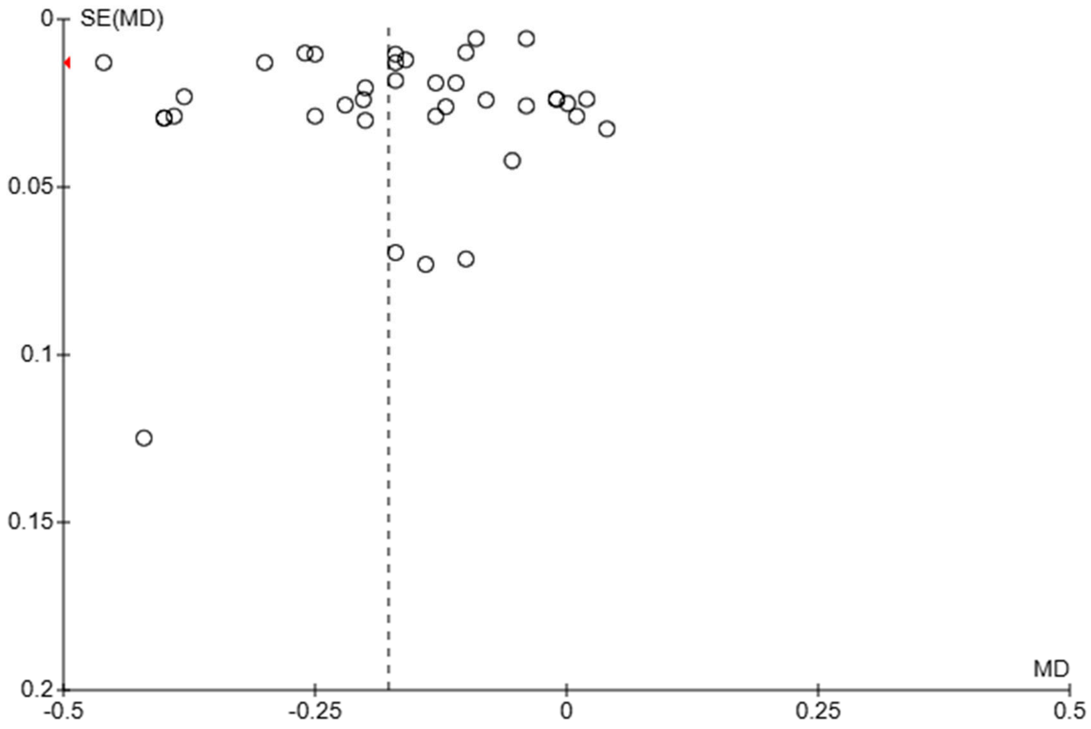

Figure S1. Funnel plot for publication bias assessment of pH values in meat products with apple pomace addition

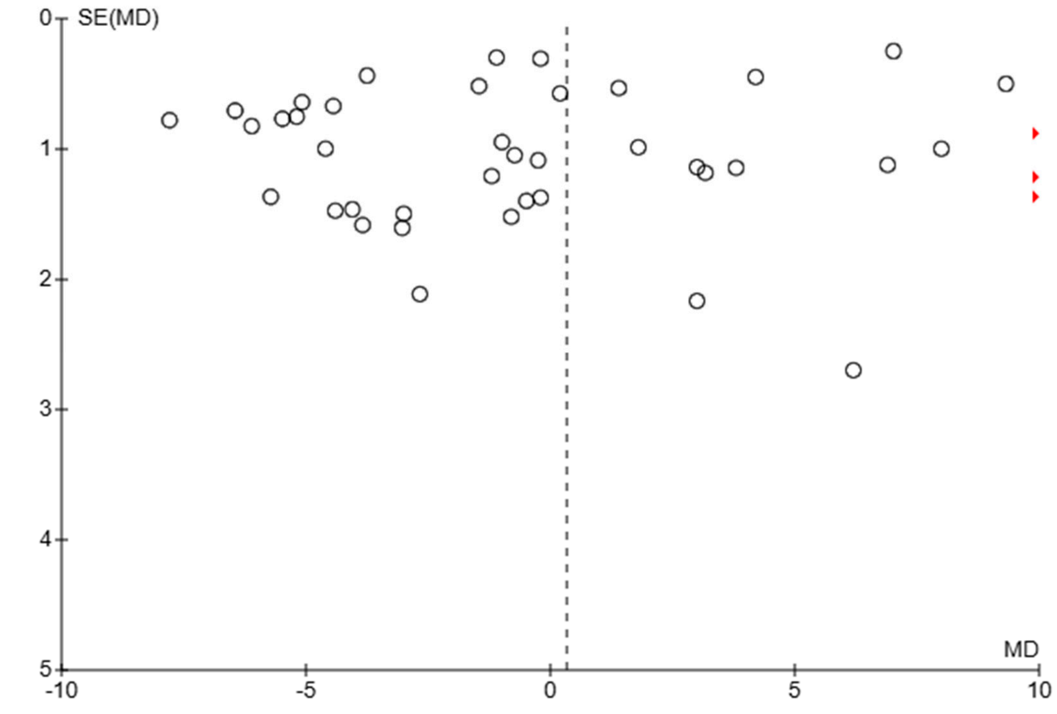

Figure S2. Funnel plot for publication bias assessment of *L values in meat products with apple pomace addition**

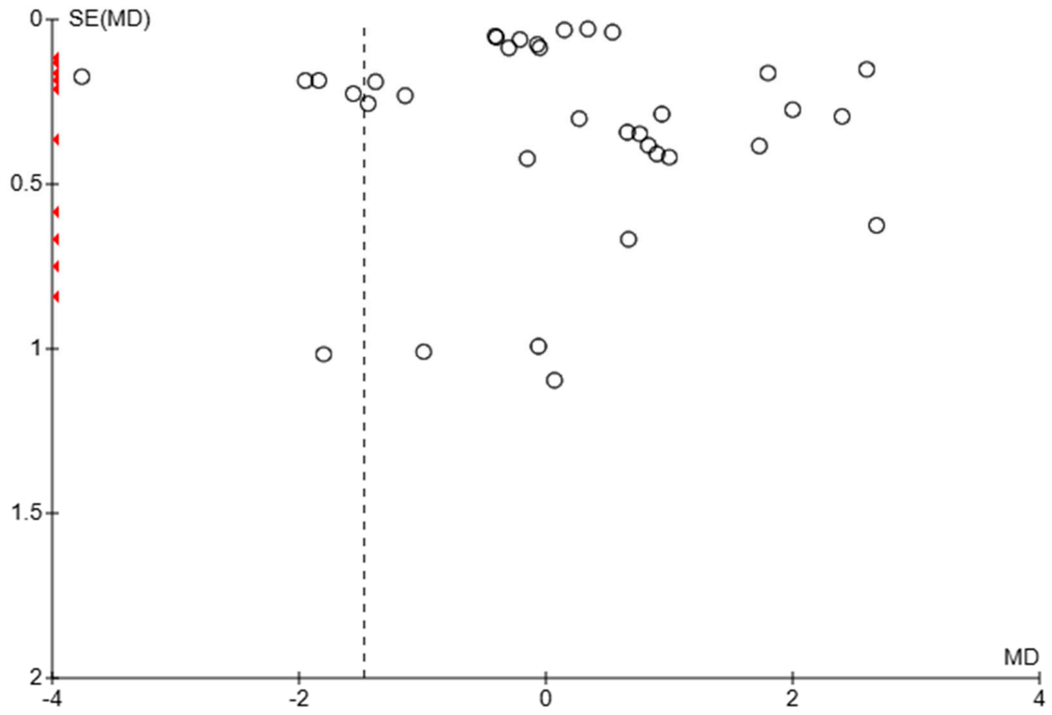

Figure S3. Funnel plot for publication bias assessment of *a values in meat products with apple pomace addition**

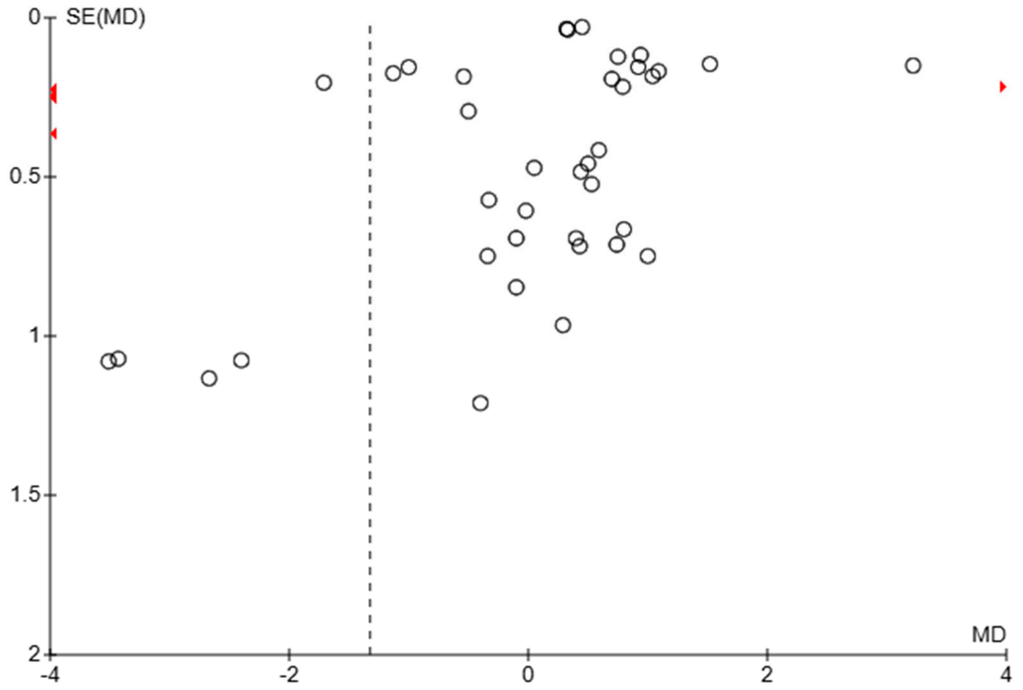

Figure S4. Funnel plot for publication bias assessment of *b values in meat products with apple pomace addition**
